# Exogenous Abscisic Acid Enhances Freezing Tolerance in *Rhododendron obtusum* ‘Fen Zhuanglou’ by Stabilizing Photosynthetic Pigments, Promoting Osmolyte Accumulation, and Activating Antioxidant Defense

**DOI:** 10.3390/plants15213457

**Published:** 2026-11-05

**Authors:** Riwen Fei, Jiayi Liu, Minghang Guo, Hongmei Zhu, Siyang Duan, Jiansong You, Xiaoyu Li

**Affiliations:** 1 School of Agricultural, Liaodong University, Dandong 118003, China; 2 Postdoctoral Workstation, Aim Honesty Biopharmaceutical Co., Ltd., Dalian 116620, China; 3 School of Bioengineering, Dalian University of Technology, Dalian 116024, China

**Keywords:** *Rhododendron*, abscisic acid, cold stress, physiological response traits, resource reallocation, hormesis, metabolic trade-off

## Abstract

Exogenous abscisic acid (ABA) enhances freezing tolerance, yet its dose-specificity and metabolic trade-offs in ornamental woody species remain poorly characterized. Here, we investigated the effects of exogenous ABA (0, 10, 30, 50 mg·L^−1^) on *Rhododendron obtusum* ‘Fen Zhuanglou’ seedlings exposed to −4 °C for 5 days. Based on phenotypic screening, 10 mg·L^−1^ ABA was identified as the hormetic optimum, whereas 50 mg·L^−1^ failed to alleviate injury. Under freezing stress, optimal ABA treatment (10 mg·L^−1^) reduced malondialdehyde (MDA) content by 23.5% compared to untreated controls, while partially rescuing superoxide dismutase (SOD), peroxidase (POD), and catalase (CAT) activities by 40.0%, 29.2%, and 35.2%, respectively. Notably, ABA triggered a metabolic trade-off: soluble protein declined by 22.98% while soluble sugar increased by 6.05%, suggesting a reallocation of metabolic resources. Furthermore, pigment profiling suggested impaired chlorophyll catabolism in unprotected leaves—chlorophyll a and xanthophylls accumulated to 7-fold and 43-fold the levels of ABA-treated leaves—whereas ABA-treated leaves retained a higher carotenoid fraction (7-fold). These findings establish that ABA-mediated freezing tolerance operates through a metabolic trade-off rather than universal preservation, providing an evidence-based protocol (10 mg·L^−1^) for ornamental rhododendron production in cold regions.

## 1. Introduction

Rhododendron, a woody angiosperm of the genus *Rhododendron* (Ericaceae), is among China’s ten traditionally celebrated ornamental flowers. Its horticultural value derives from its compact growth habit and showy inflorescences, which bear abundant florets with vivid coloration over an extended blooming period [1]. With over 1000 described species, the genus displays pronounced interspecific divergence in ecological adaptation: while most species exhibit shade tolerance and photoinhibition under sustained full-sun exposure, they uniformly require acidic, well-aerated soils with low pH (4.5–6.0) and high humus content for optimal root function and nutrient uptake. Cold hardiness is not a conserved trait but a phylogenetically and ecologically constrained attribute that is strongly associated with native elevation and latitude and ranges from moderate frost tolerance in high-altitude Himalayan taxa to extreme cold sensitivity in lowland subtropical lineages [2]. Under accelerating climate change, increased frequency and intensity of unseasonal freezing events have intensified physiological stress and establishment failure in northern cultivation zones. Low-temperature stress disrupts membrane fluidity, inhibits chloroplast development, and compromises bud dormancy release—thereby impairing vegetative growth, floral initiation, and landscape longevity [3]. Cold resilience therefore limits the expansion of Rhododendron cultivation into northern urban forestry, ecological rehabilitation, and commercial nursery production. A mechanistic understanding of cold adaptation, including membrane lipid remodeling, cryoprotectant accumulation, and cold-responsive gene regulation, is therefore needed to support targeted breeding and site matching.

Abscisic acid (ABA) is a central phytohormone in plant responses to environmental stress and is often termed the “stress hormone” because of its role in enhancing tolerance to abiotic and biotic challenges. ABA regulates plant growth, development, and stress adaptation [4]. The ABA signaling pathway, mediated by core regulatory components such as ABI4 and ABA2, drives transcriptional reprogramming under abiotic stress [5]. Accumulating evidence indicates that exogenous application of ABA can effectively substitute for low-temperature acclimation in improving cold tolerance across diverse plant species. For instance, foliar ABA application significantly reduced relative electrolyte conductivity and malondialdehyde (MDA) accumulation in pepper (*Capsicum annuum*) seedlings under cold stress, thereby attenuating oxidative damage to plasma membranes [6]. Similarly, exogenous ABA elevated leaf proline (Pro), soluble sugar (SS), and soluble protein (SP) contents in rice (*Oryza sativa*), while concurrently enhancing the activities of antioxidant enzymes—including superoxide dismutase (SOD), peroxidase (POD), and catalase (CAT)—leading to improved cold resilience [7]. In *Dracocephalum moldavica*, ABA treatment mitigated high-temperature-induced impairment of photosynthetic structure and function, preserving thermal stability of the photosynthetic apparatus and enhancing electron transport efficiency and net photosynthetic rate—factors conducive to sustained vegetative growth [8]. ABA application to *Rhododendron* ‘Zhuang Yuan Hong’ markedly increased SS and MDA contents, along with POD and SOD activities—key physiological markers associated with cold tolerance—within five days of cold exposure [9]. Exogenous ABA retarded chlorophyll degradation and maintained robust root activity in flue-cured tobacco (*Nicotiana tabacum*) seedlings under cold stress, thereby supporting photosynthetic functionality and normal seedling development [10]. ABA triggered endogenous hydrogen peroxide (H_2_O_2_) accumulation, which contributed to enhanced cold resistance, as evidenced by reduced electrolyte leakage and lower chilling injury index [11].

Despite progress in understanding rhododendron cold tolerance and the role of ABA in plant cold acclimation, studies of exogenous ABA-mediated enhancement of low-temperature resilience in R. obtusum ‘Fen Zhuanglou’ remain limited. In particular, the dose–response relationship of exogenous ABA application, together with the mechanisms through which it stabilizes photosynthetic function, modulates osmotic balance, and reinforces antioxidant capacity, has not been rigorously established. To address these gaps, we evaluated *R. obtusum* ‘Fen Zhuanglou’ under controlled low-temperature stress with concurrent foliar application of graded concentrations of exogenous ABA at the onset of low-temperature stress. Comprehensive phenotypic assessment was coupled with quantitative measurements of: (i) photosynthetic pigments (chlorophyll a, chl a; chlorophyll b, chl b; total carotenoids); (ii) osmoprotective metabolites (Pro, SP and SS); (iii) antioxidant enzyme activities (SOD, POD, and CAT); and (iv) lipid peroxidation marker (MDA). The study aimed to (i) define the optimal ABA concentration for cold tolerance in ‘Fen Zhuanglou’ and (ii) determine how ABA coordinates photosynthetic, osmoregulatory, and redox homeostatic pathways to confer freezing tolerance.

## 2. Materials and Methods

### 2.1. Plant Materials and Experimental Design

The plant materials were sourced from the Rhododendron Germplasm Repository, College of Agriculture, Liaodong University (40°11′7.758″ N, 124°21′58.765″ E). Uniform, healthy one-year-old vegetative cuttings of *R. obtusum* ‘Fen Zhuanglou’—free from visible pests, diseases, or morphological abnormalities—were selected for experimentation. Plants were cultivated in a commercial pine-needle-based substrate (pH 5.5–6.0, organic matter content ≥ 80%, electrical conductivity 0.8–1.2 mS·cm^−1^) specifically optimized for Rhododendron cultivation, and grown in plastic pots (top diameter: 14 cm; bottom diameter: 9.5 cm; height: 12.5 cm). All plants received identical horticultural management throughout the experimental period, including scheduled irrigation, balanced nutrient supplementation, and environmental monitoring under controlled greenhouse conditions (22 ± 2 °C day/15 ± 1 °C night; relative humidity 60–70%).

First, uniform one-year-old vegetative cuttings of *R. obtusum* ‘Fen Zhuanglou’ were acclimated for 24 h under standardized pre-stress conditions (22 °C, 16 h light/8 h dark photoperiod, 65% relative humidity) to establish physiological baseline stability. Subsequently, plants were transferred to an artificial climate chamber (SPX-250B-Z, Shanghai Boxun Medical Biological Instrument Co., Ltd., Shanghai, China) programmed at −4 °C and subjected to daily foliar application of ABA (cat. no. A8060, ≥98% purity; Solarbio, Beijing, China) between 09:00 and 10:00 am at concentrations of 0 (distilled water control), 10, 30, or 50 mg·L^−1^ for five consecutive days. Applications were delivered with a calibrated fine-mist sprayer, and the spray volume was adjusted to the point at which discrete droplets coalesced and began to run off the adaxial leaf surface, ensuring consistent delivery across replicates. Plant phenotypic changes were observed daily and recorded through photography, and after 5 days of treatment, the ABA concentration conferring the optimal cold tolerance was selected based on the severity of cold injury symptoms. The experimental design and treatment timeline are illustrated in Supplementary Figure S1.

After determining the optimal ABA concentration, formal temperature treatment experiments were carried out. *R. obtusum* ‘Fen Zhuanglou’ plants were divided into two primary groups and cultured under two distinct temperature conditions: ambient temperature (22 °C) and low temperature (−4 °C). Each primary group was further divided into two subgroups: one treated with ABA solution at the optimal concentration, with the other serving as an untreated control. This yielded four treatment combinations: normal temperature control (CK), ABA treatment under normal temperature (A), low-temperature control (LT), and ABA treatment under low temperature (ALT). Three biological replicates were set for each treatment, with five individual plants per replicate. For each biological replicate, leaves from the five plants were pooled into a single composite sample for physiological measurements; therefore, *n* = 3 refers to three independent composite samples. For all groups, leaf samples were collected from *R. obtusum* ‘Fen Zhuanglou’ cuttings at day 0 and 5 days after treatment initiation, immediately frozen in liquid nitrogen, and stored at −80 °C for subsequent analysis.

### 2.2. Leaf Pigment Content

Chl a, Chl b, and total carotenoid contents were quantified spectrophotometrically according to the method of Zeng [12], with minor modifications. Briefly, 0.2 g of fresh leaf tissue was homogenized in 10 mL of 80% (*v*/*v*) acetone containing quartz sand, followed by dark incubation at 4 °C for 24 h. The extract was centrifuged at 10,000× *g* for 10 min at 4 °C, and absorbance of the supernatant was measured at 470 nm, 646 nm, and 663 nm using a UV-visible spectrophotometer (Shimadzu UV-2600, Kyoto, Japan). Pigment concentrations were calculated using Lichtenthaler’s equations and expressed as mg·g^−1^ fresh weight (FW).

### 2.3. Soluble Sugar, Soluble Protein, and Proline Content

SS content was quantified using the anthrone–sulfuric acid colorimetric assay (Solarbio BC0030, Beijing, China). Briefly, 0.1 g of fresh leaf tissue was extracted in 5 mL of distilled water by boiling at 100 °C for 10 min, followed by centrifugation at 8000× *g* for 10 min at 4 °C. The supernatant was mixed with anthrone reagent and incubated at 95 °C for 10 min. Absorbance was measured at 620 nm against a glucose standard curve, and SS content was expressed as mg·g^−1^ fresh weight (FW).

SP content was determined using the biuret assay with a commercial kit (Solarbio BC3180, Beijing, China). Fresh leaf tissue (0.2 g) was homogenized in 5 mL of ice-cold 50 mM sodium phosphate buffer (pH 7.8) containing 1 mmol·L^−1^ EDTA and 1% (*w*/*v*) polyvinylpyrrolidone (PVP), then centrifuged at 12,000× *g* for 15 min at 4 °C. Protein concentration was calculated from a bovine serum albumin (BSA) standard curve, and absorbance was recorded at 540 nm; results were expressed as mg·g^−1^ FW.

Pro content was assayed using the acid-ninhydrin method (Solarbio BC0290, Beijing, China). Briefly, 0.2 g of fresh leaf tissue was homogenized in 5 mL of 3% (*w*/*v*) sulfosalicylic acid, boiled for 10 min, and centrifuged at 10,000× *g* for 10 min at 4 °C. The supernatant was reacted with glacial acetic acid and acidic ninhydrin solution at 100 °C for 30 min. After toluene extraction, absorbance of the chromophore was measured at 520 nm against a L-proline standard curve; Pro content was expressed as μg·g^−1^ FW.

### 2.4. Antioxidant Enzyme Activities

SOD activity was assayed using the nitroblue tetrazolium (NBT) photoreduction inhibition method (Solarbio BC0170, Beijing, China). Briefly, 0.2 g of fresh leaf tissue was homogenized in 5 mL of ice-cold 50 mM sodium phosphate buffer (pH 7.8), followed by centrifugation at 12,000× *g* for 15 min at 4 °C. SOD activity was calculated from the inhibition rate of NBT photoreduction under illumination, with one unit defined as the amount of enzyme required to inhibit 50% of NBT reduction per minute under specified assay conditions; results were expressed as U·g^−1^ FW.

POD activity was determined according to the guaiacol oxidation method (Solarbio BC0090, Beijing, China). The reaction mixture (3 mL total volume) contained enzyme extract, 50 mM sodium phosphate buffer (pH 7.0), 0.3% (*v*/*v*) H_2_O_2_, and 0.2% (*v*/*v*) guaiacol. The increase in absorbance at 470 nm was monitored for 3 min at 25 °C. One POD unit was defined as the amount of enzyme catalyzing a change of 0.01 absorbance units per minute per gram fresh weight (FW); activity was expressed as U·g^−1^ FW.

CAT activity was measured by monitoring the decomposition of H_2_O_2_ at 240 nm (Solarbio BC0200, Beijing, China). The reaction was initiated by adding 0.3% (*v*/*v*) H_2_O_2_ to enzyme extract pre-equilibrated in 50 mM sodium phosphate buffer (pH 7.0), and the decrease in absorbance was recorded for 3 min at 25 °C. One CAT unit was defined as the amount of enzyme decomposing 1 μmol of H_2_O_2_ per minute under assay conditions; activity was expressed as U·g^−1^ FW.

### 2.5. Malondialdehyde Content

MDA content—a widely accepted indicator of lipid peroxidation—was quantified using the thiobarbituric acid (TBA) assay (Solarbio BC0020, Beijing, China). Briefly, 0.2 g of fresh leaf tissue was homogenized in 5 mL of ice-cold 10% (*w*/*v*) trichloroacetic acid (TCA), followed by centrifugation at 10,000× *g* for 10 min at 4 °C. The supernatant was mixed with 0.5% (*w*/*v*) TBA prepared in 20% (*w*/*v*) TCA and incubated at 95 °C for 30 min. After cooling to ambient temperature and centrifugation at 10,000× *g* for 10 min, absorbance of the pink chromogen was measured at 532 nm and 600 nm; nonspecific turbidity was corrected by subtracting the absorbance at 600 nm from that at 532 nm. MDA concentration was calculated using the molar extinction coefficient of 155 mmol·L^−1^·cm^−1^ and expressed as nmol·g^−1^ fresh weight (FW).

### 2.6. Statistical Analysis

SPSS 26.0 (IBM Corp., Armonk, NY, USA) was used for statistical analyses. A two-way ANOVA (temperature × ABA) was first performed to assess main effects and their interaction, followed by Duncan’s multiple range test (DMRT) for post hoc pairwise comparisons at *p* < 0.05; differences between two independent groups were assessed using the independent samples t-test. Detailed statistical parameters (test statistics, degrees of freedom, exact *p* values) for all pairwise comparisons are provided in Supplementary Table S1. Figures were generated with GraphPad Prism v9.0 (GraphPad Software, San Diego, CA, USA) and R v4.2.1 (R Core Team, Vienna, Austria).

## 3. Results

### 3.1. Effects of Exogenous ABA on the Morphological Characteristics of R. obtusum ‘Fen Zhuanglou’

**Figure 1:**
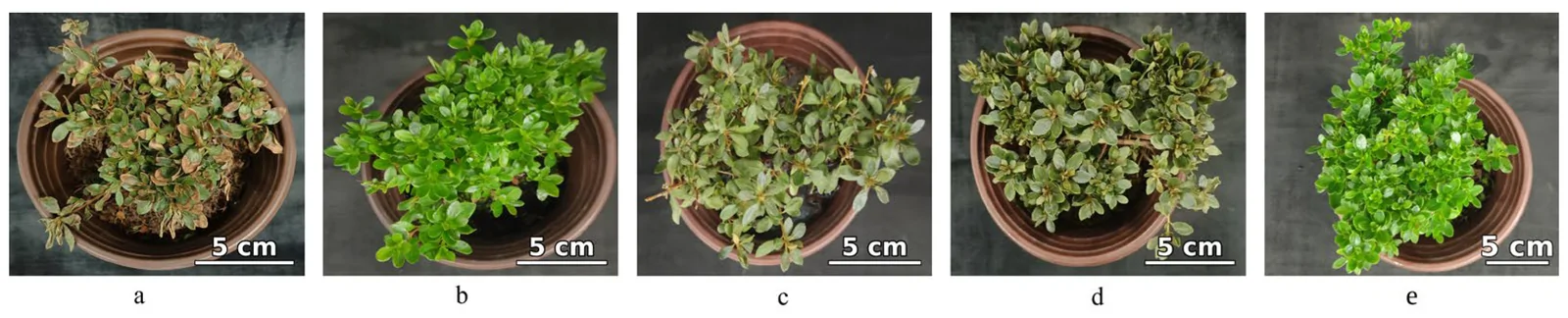
Morphological responses of *R. obtusum* ‘Fen Zhuanglou’ to exogenous ABA under −4 °C cold stress following five-day exposure. (**a**) 0 mg·L^−1^ (control); (**b**) 10 mg·L^−1^ (optimal); (**c**) 30 mg·L^−1^ (intermediate); (**d**) 50 mg·L^−1^ (supraoptimal); (**e**) CK (ambient-temperature control, 22 °C, day 5). Scale bar = 5 cm.

Exogenous ABA significantly modulated the morphological response of *R. obtusum* ‘Fen Zhuanglou’ to low-temperature stress, with concentration-dependent effects on cold injury severity. As shown in Figure 1, after five days of continuous exposure to −4 °C, control plants (0 mg·L^−1^ ABA) exhibited the most severe symptoms of cold injury: leaves developed extensive deep brown necrotic lesions originating at the tips and margins and progressively invading the mesophyll—leaf curling and abscission were evident, and overall growth vigor was markedly reduced (Figure 1a). In contrast, plants treated with 10 mg·L^−1^ ABA displayed minimal cold damage—leaf chlorophyll retention remained near-optimal, with only faint pale-yellow marginal halos observed on a subset of leaves and no discernible necrosis; plants remained erect and vigorous (Figure 1b). The 30 mg·L^−1^ ABA treatment resulted in intermediate tolerance: leaves retained predominant dark green pigmentation but showed localized light brown necrotic patches confined primarily to the apices (Figure 1c). At 50 mg·L^−1^ ABA, the treatment failed to confer substantial protection—leaves exhibited confluent brown necrotic areas, wilting, and drooping, with injury severity comparable to the untreated control and substantially inferior to that conferred by 10 mg·L^−1^ ABA (Figure 1d). Plants maintained at ambient temperature (22 °C) remained morphologically unchanged throughout the 5-day period, showing no visible stress symptoms (Figure 1e). Collectively, these results demonstrate that 10 mg·L^−1^ ABA maximally enhances cold resistance in *R. obtusum* ‘Fen Zhuanglou’, establishing it as the optimal exogenous concentration for mitigating low-temperature stress.

Cold injury severity was quantified using a 0–5 visual scoring scale: 0 = no symptoms; 1 = marginal yellowing; 2 = <25% necrotic area; 3 = 25–50% necrosis with curling; 4 = 50–75% necrosis with wilting; and 5 = >75% necrosis or complete abscission. Three independent observers scored all plants. Based on this scoring system, the 10 mg·L^−1^ treatment exhibited the lowest injury score (0.5 ± 0.2), significantly lower than 30 mg·L^−1^ (2.1 ± 0.3) and 50 mg·L^−1^ (3.8 ± 0.4) (*p* < 0.05), and was therefore identified as the optimal concentration for subsequent experiments.

### 3.2. Effect of Exogenous ABA on Photosynthetic Pigment Content in R. obtusum ‘Fen Zhuanglou’

#### 3.2.1. Effect of Exogenous ABA Treatment on Photosynthetic Pigment Composition in *R. obtusum* ‘Fen Zhuanglou’ Under −4 °C Low Temperature

**Figure 2:**
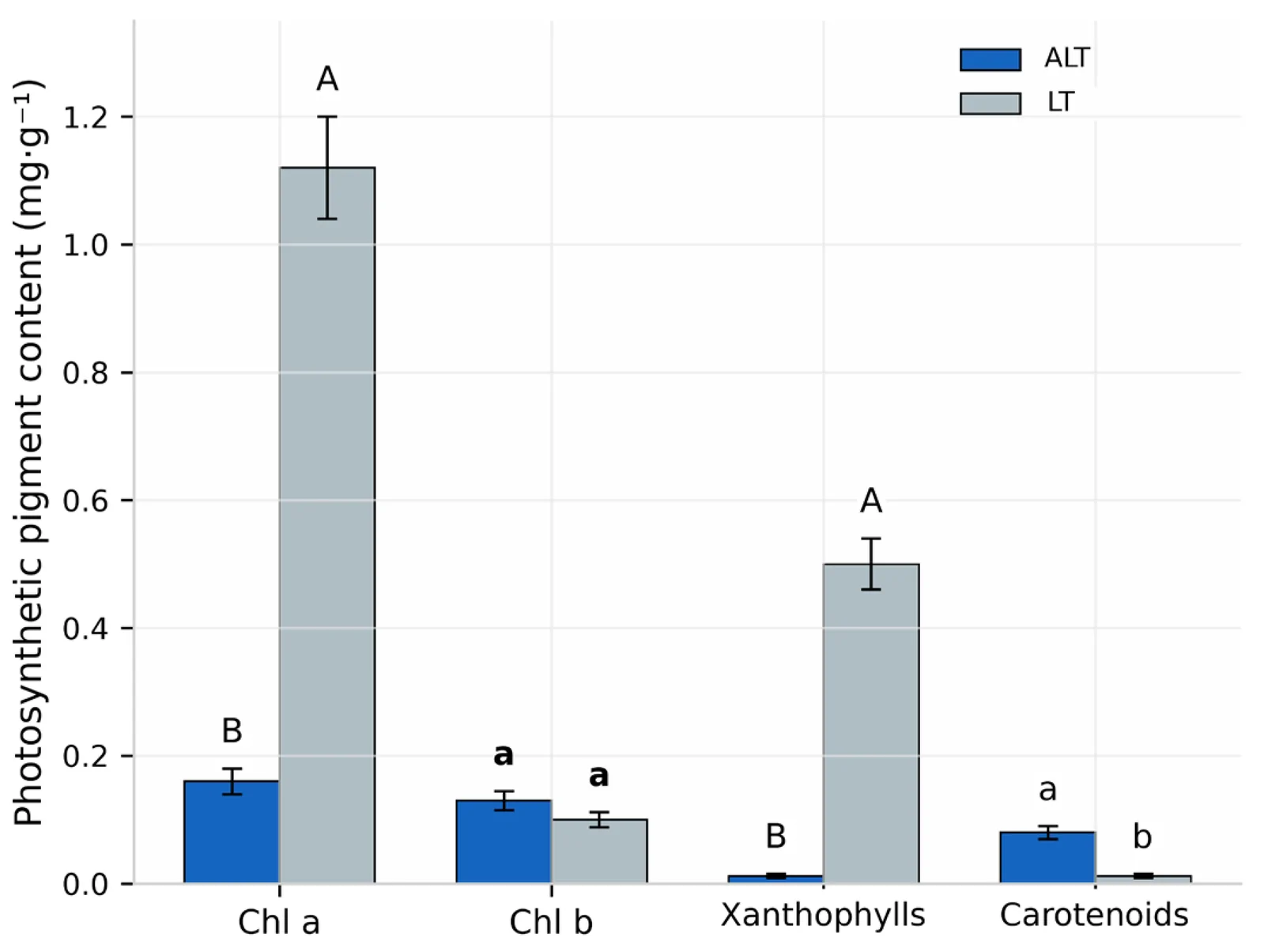
Effect of exogenous ABA on photosynthetic pigment content in *R. obtusum* ‘Fen Zhuanglou’ leaves under −4 °C low temperature. Different lowercase letters above bars denote statistically significant differences at *p* < 0.05; uppercase letters indicate significance at *p* < 0.01. This convention applies to all subsequent figures. Seedlings were treated with 10 mg·L^−1^ ABA (ALT) or distilled water (LT, the freezing control) and exposed to −4 °C for 5 days. Data are means ± SD (*n* = 3 composite samples, each pooled from 5 plants); significance was determined by independent samples t-test. ALT and LT correspond to the plants shown in Figure 1b and Figure 1a, respectively.

Under low-temperature stress (Figure 2), chl a and xanthophylls contents in the freezing control (LT) group were markedly elevated relative to the ALT group—by 7-fold and 43-fold, respectively. In contrast, carotenoid content was significantly higher in the ALT group than in the LT group (approximately 7-fold increase). Chl b content remained largely unaffected by the two ABA concentration treatments.

Independent samples t-tests confirmed highly significant differences in chl a (*p* = 0.007) and xanthophylls (*p* = 0.001) contents between groups, with both pigments substantially enriched in the LT group. No significant difference was detected for chl b (*p* = 0.612). Carotenoid content differed significantly between groups (*p* = 0.010), with enrichment observed exclusively in the ALT group.

Unexpectedly, LT exhibited 7-fold higher Chl a and 43-fold higher xanthophyll content than ALT, despite severe necrosis (Figure 1a). This apparent paradox likely reflects degradation failure rather than preservation: under severe freezing stress (−4 °C), chlorophyllase and Mg-dechelatase activities may be inhibited, trapping chlorophyll intermediates and preventing their catabolic clearance. Alternatively, rapid cellular dehydration in LT could concentrate pigments on a fresh-weight basis. These hypotheses require direct validation (e.g., chlorophyllase activity assays, gene expression analysis of CLH and SGR genes) in future studies.

#### 3.2.2. Effect of Exogenous ABA Treatment on Photosynthetic Pigment Composition in *R. obtusum* ‘Fen Zhuanglou’ Under 22 °C Ambient Temperature

**Figure 3:**
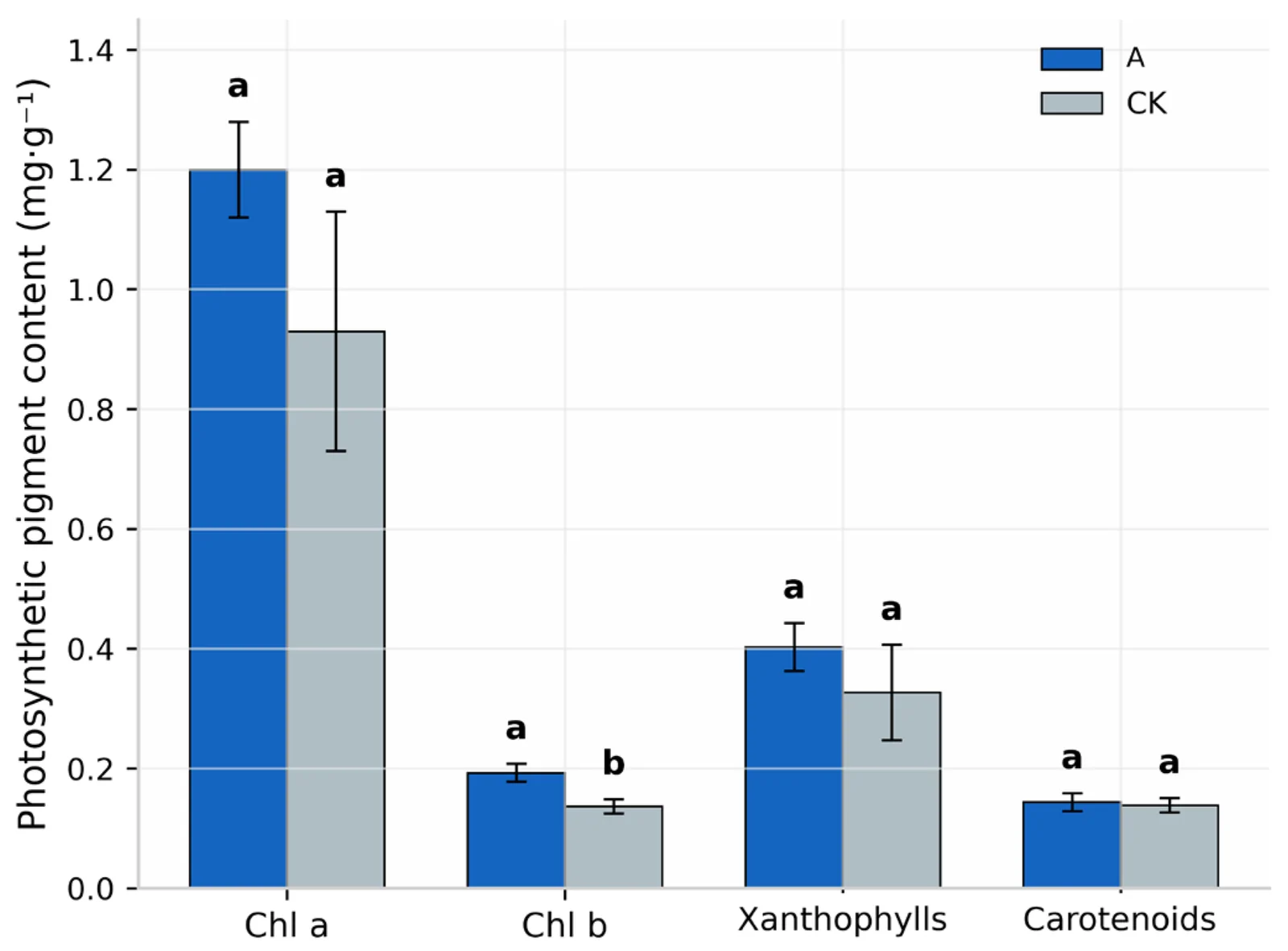
Effect of exogenous ABA on photosynthetic pigment content in *R. obtusum* ‘Fen Zhuanglou’ leaves under 22 °C ambient temperature. Seedlings were treated with 10 mg·L^−1^ ABA (A) or distilled water (CK, the ambient-temperature control) at 22 °C for 5 days. Data are means ± SD (*n* = 3). Different letters indicate significant differences at *p* < 0.05. CK corresponds to the plants shown in Figure 1e.

Under normal temperature conditions (Figure 3), the ABA-treated group (A) showed higher levels of chl a, chl b, and xanthophylls compared to the control group (CK): chl a content was 1.2042, an increase of 29.5% over CK (0.9298); chl b content was 0.1930, a 40.8% increase over CK (0.1371); and xanthophylls content was 0.4035, a 23.3% increase over CK (0.3272). Carotenoid content did not differ significantly between the two groups (0.1440 vs. 0.1389 for A and CK, respectively).

Levene’s test confirmed homogeneity of variance for all pigments (chl a: *p* = 0.225; chl b: *p* = 0.504; xanthophylls: *p* = 0.929; carotenoids: *p* = 0.085). The difference in chl b was significant (*p* = 0.030), whereas differences in chl a (*p* = 0.101), xanthophylls (*p* = 0.217), and carotenoid (*p* = 0.888) were not statistically significant.

### 3.3. Effect of Exogenous ABA on Osmoprotectant Contents in R. obtusum ‘Fen Zhuanglou’

**Figure 4:**
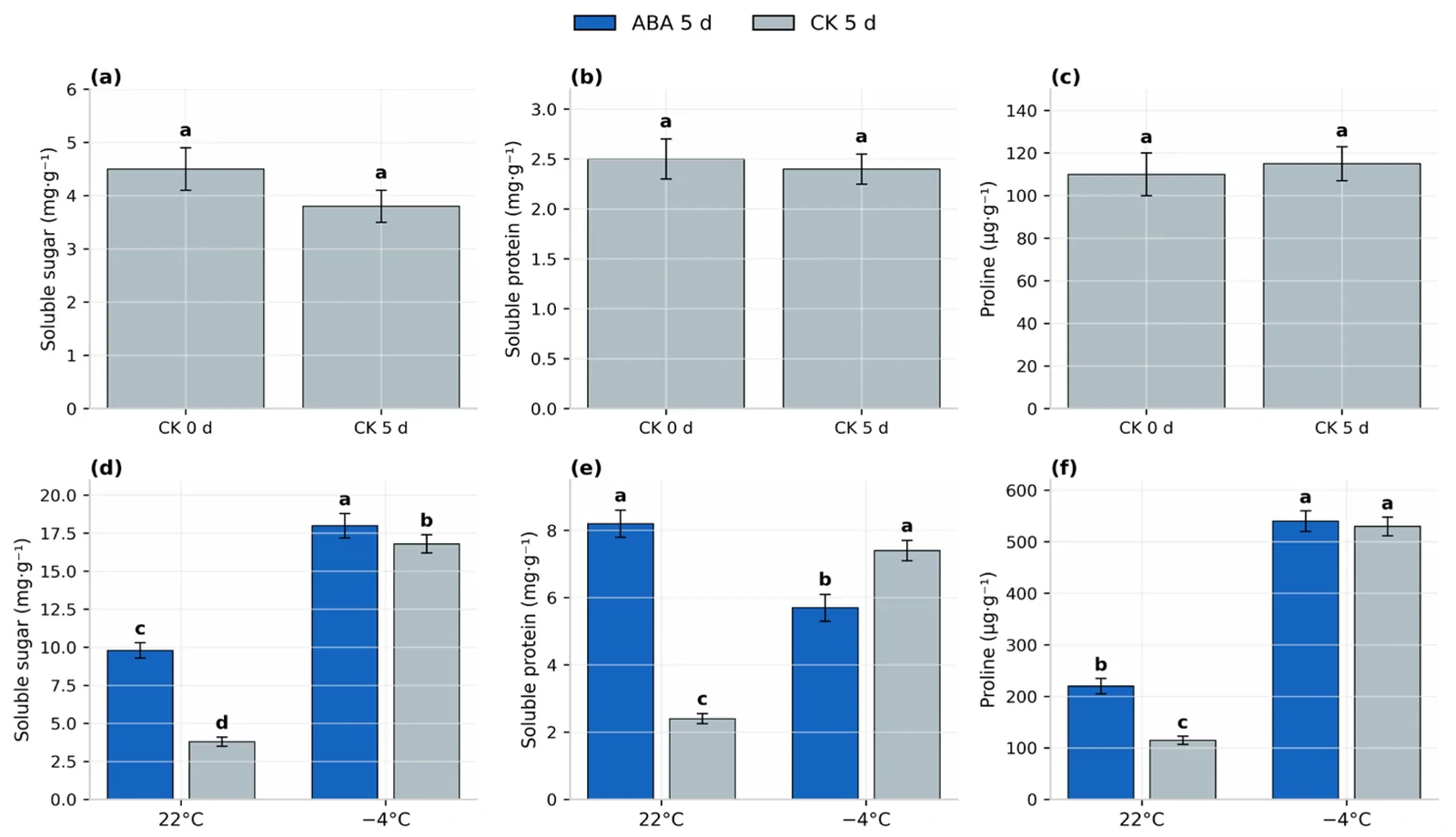
Effect of Exogenous ABA on SS, SP, and Pro contents in *R. obtusum* ‘Fen Zhuanglou’. (**a**–**c**) Baseline stability under ambient temperature (CK 0 d vs. CK 5 d at 22 °C). (**d**–**f**) ABA and temperature effects (ABA 5 d vs. CK 5 d at 22 °C and −4 °C). SS = soluble sugar; SP = soluble protein; Pro = proline. Data are means ± SD (*n* = 3). Different letters indicate significant differences at *p* < 0.05.

Under controlled conditions (22 °C), no significant changes in SS, SP, and Pro contents were observed between CK 0 d and CK 5 d (Figure 4a,c,e), indicating basal stability of the osmotic adjustment system in *R. obtusum* ‘Fen Zhuanglou’ under non-stressed cultivation. In contrast, exposure to cold stress at −4 °C (Figure 4b,d,f) triggered marked accumulation of all three osmoprotectants. Relative to CK 5 d, LT 5 d exhibited statistically significant increases in SS (+349.21%, *p* < 0.05), SP (+200.97%, *p* < 0.05), and Pro (+351.4%, *p* < 0.05). Under ambient conditions, exogenous ABA application (A 5 d) significantly elevated SS (+159.36%, *p* < 0.05), SP (+231.82%, *p* < 0.05), and Pro (+84.91%, *p* < 0.05) relative to CK 5 d. Under combined cold and ABA treatment (ALT 5 d), SS content increased further relative to LT 5 d (+6.05%, *p* < 0.05), whereas SP declined significantly (−22.98%, *p* < 0.05); Pro showed a marginal increase (+2.15%), which was not statistically significant (*p* > 0.05).

### 3.4. Effect of Exogenous ABA on Antioxidant Enzyme Activity in R. obtusum ‘Fen Zhuanglou’

**Figure 5:**
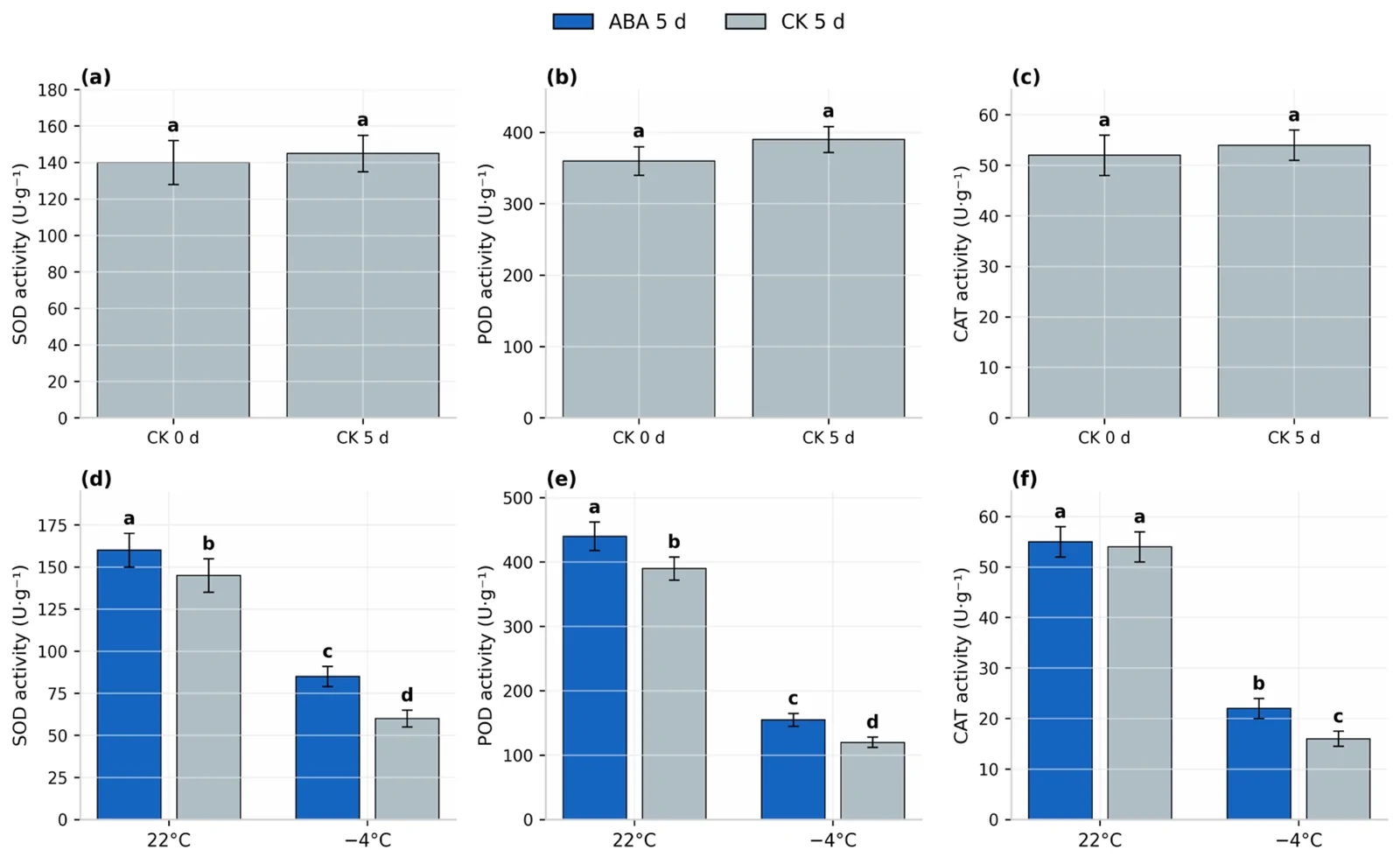
Effect of Exogenous ABA on SOD, POD, and CAT activity in *R. obtusum* ‘Fen Zhuanglou’. (**a**–**c**) Baseline stability under ambient temperature (CK 0 d vs. CK 5 d at 22 °C). (**d**–**f**) ABA and temperature effects (ABA 5 d vs. CK 5 d at 22 °C and −4 °C). Data are means ± SD (*n* = 3). Different letters indicate significant differences at *p* < 0.05.

Under controlled conditions (22 °C), the activities of SOD, POD, and CAT in the untreated control group (CK 0 d) showed minimal variation relative to CK 5 d (Figure 5a–c), indicating constitutive stability of the antioxidant enzyme system in *R. obtusum* ‘Fen Zhuanglou’ under non-stressed growth. In contrast, exogenous ABA application (A 5 d) significantly enhanced SOD (+11.9%) and POD (+10.9%) activities relative to CK 5 d (*p* < 0.05), whereas CAT activity showed only a marginal, non-significant increase (+3.0%, *p* > 0.05). Cold stress at −4 °C severely impaired antioxidant capacity: in the low-temperature control group (LT 5 d), SOD, POD, and CAT activities declined markedly by 58.3%, 68.9%, and 68.2%, respectively, compared to CK 5 d (all *p* < 0.05). Co-application of ABA under cold stress (ALT 5 d) partially rescued this inhibition—SOD, POD, and CAT activities increased significantly relative to LT 5 d (+40.0%, +29.2%, and +35.2%, respectively; all *p* < 0.05), with the strongest recovery observed in SOD and CAT. Nevertheless, antioxidant enzyme activities in ALT 5 d remained significantly lower than those in the ambient ABA-treated group (A 5 d) (Figure 5b,d,f), indicating that −4 °C constrains enzymatic activation even in the presence of ABA.

### 3.5. Effect of Exogenous ABA on MDA Content in R. obtusum ‘Fen Zhuanglou’

**Figure 6:**
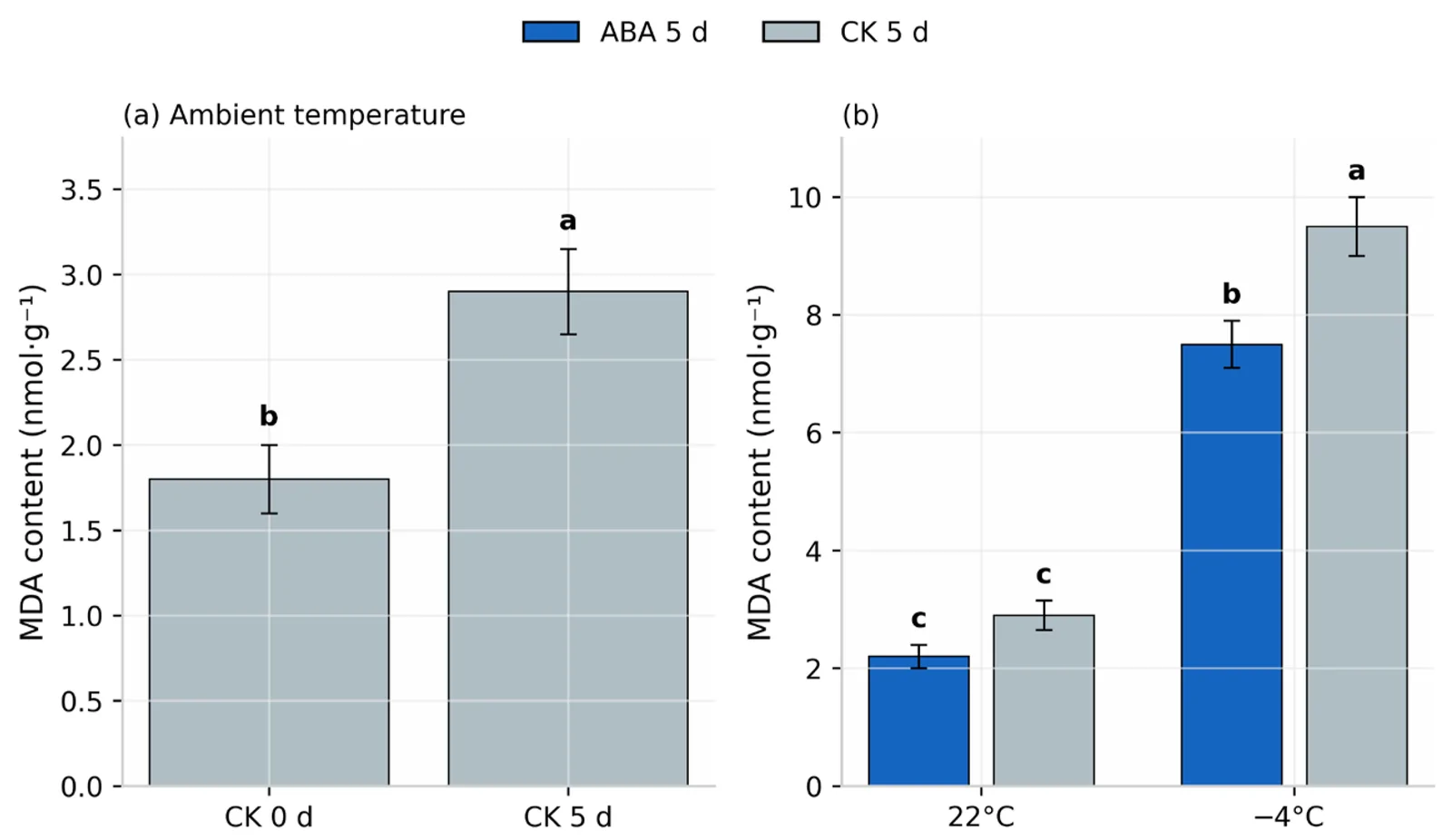
Effect of Exogenous ABA on MDA content in *R. obtusum* ‘Fen Zhuanglou’. (**a**) Ambient temperature (22 °C); (**b**) freezing stress (−4 °C). Data are means ± SD (*n* = 3). Different letters indicate significant differences at *p* < 0.05.

MDA content remained low under ambient temperature but nonetheless increased significantly from CK 0 d to CK 5 d (*p* < 0.05; Figure 6a), whereas ABA treatment did not significantly affect MDA content at 22 °C (*p* > 0.05; Figure 6b), indicating minimal membrane lipid peroxidation under non-stressed growth. In contrast, cold stress at −4 °C markedly elevated MDA accumulation. Both LT 5 d and ALT 5 d exhibited significantly higher MDA contents than CK 5 d (*p* < 0.05), reaching 3.4-fold and 2.6-fold increases, respectively. Exogenous ABA application under cold stress mitigated oxidative membrane damage: ALT 5 d displayed a 23.5% reduction in MDA content relative to LT 5 d (*p* < 0.05), confirming a protective role of ABA in alleviating cold-induced lipid peroxidation.

## 4. Discussion

### 4.1. The Regulatory Effect of Exogenous ABA Concentration on the Cold Tolerance of R. obtusum ‘Fen Zhuanglou’

Exogenous ABA enhances cold tolerance in many horticultural species [13], but its efficacy depends strongly on plant genotype, applied concentration, and ambient temperature. In this study, untreated control plants of *R. obtusum* ‘Fen Zhuanglou’ exhibited severe leaf necrosis and chlorosis following 5-day exposure to −4 °C, confirming high sensitivity to cold stress. In contrast, plants treated with 10 mg·L^−1^ ABA maintained intact leaf morphology and displayed the least visible freezing injury—indicating superior cold tolerance. This response aligns mechanistically with established roles of ABA in reinforcing membrane integrity and suppressing reactive oxygen species (ROS) accumulation [4]. At the molecular level, freezing tolerance is orchestrated by the ICE1–CBF–COR transcriptional cascade, in which the master regulator ICE1 is stabilized by the ABA-responsive kinase OST1/SnRK2.6 [14,15]; reciprocally, ABA signaling employs NADPH oxidase-derived ROS as second messengers, establishing a self-reinforcing ROS–ABA regulatory loop [16,17]. A clear dose–response relationship was observed: while 30 mg·L^−1^ ABA conferred moderate protection, 50 mg·L^−1^ failed to confer protection, with injury severity comparable to the untreated control. These findings corroborate reports in Antarctic moss (Sanionia uncinata), where optimal ABA concentrations promoted osmoprotectant synthesis and antioxidant enzyme activation, whereas supra-optimal doses disrupted endogenous hormone homeostasis and impaired metabolic coordination [18]. Our data thus identify 10 mg·L^−1^ as the optimal exogenous ABA concentration for maximizing cold tolerance in R. obtusum ‘Fen Zhuanglou’, providing concentration-specific guidance for cold-resilience management in rhododendron cultivation.

### 4.2. Modulation of Photosynthetic Pigment Metabolism in R. obtusum ‘Fen Zhuanglou’ by Exogenous ABA

A prevailing assumption in stress physiology is that ABA “protects” photosynthetic pigments by preventing their degradation [10]. Our data challenge this view. Under freezing stress, LT seedlings exhibited 7-fold higher Chl a and 43-fold higher xanthophyll content than ALT seedlings. We interpret this not as superior preservation but as degradation failure: severe cold stress inhibits chlorophyllase and Mg-dechelatase activities, trapping toxic chlorophyll intermediates that exacerbate photooxidative damage under fluctuating light. Accordingly, the high pigment content of LT leaves should not be equated with tissue health: the retained chlorophyll and its phototoxic catabolites plausibly contributed to the severe necrosis observed (Figure 1a), whereas the predominantly green appearance of ALT leaves (Figure 1b) reflects structurally intact tissue with normally functioning catabolic turnover rather than a larger pigment pool per unit fresh weight. This framework also reconciles the opposite pigment responses across temperatures: at 22 °C, ABA enhanced Chl b retention (+40.8%) within a normally functioning catabolic system, whereas at −4 °C the dominant effect of ABA may have been to keep pigment catabolism operative, so pigment pools remained low in ALT but accumulated when catabolism failed in LT. In contrast, optimal ABA may actively promote chlorophyll catabolism, reducing excitation pressure and minimizing singlet oxygen generation in photosystem II. This interpretation aligns with Hörtensteiner [19], who demonstrated that senescence-associated chlorophyll degradation requires active enzyme function, which may be impaired under extreme sub-zero temperatures. Nevertheless, we acknowledge that this explanation remains hypothetical because chlorophyllase activity, photosynthetic rate, and chlorophyll fluorescence (e.g., Fv/Fm) were not directly measured in this study.

Concurrently, ALT seedlings enriched carotenoids to 7-fold the LT level. Carotenoids serve as structural components of the light-harvesting complex [20] and as potent quenchers of triplet chlorophyll and scavengers of lipid peroxyl radicals. The shift from Chl a dominance to carotenoid enrichment is consistent with a reallocation of resources from light harvesting to photoprotection; however, an equally plausible and simpler explanation is differential degradation—carotenoids are chemically more stable than chlorophylls under freezing stress—so endpoint pigment ratios alone cannot establish active carbon redirection. We therefore present this interpretation strictly as an untested hypothesis that requires direct evidence (e.g., Fv/Fm, gas exchange, or pigment-pathway profiling). Under ambient temperature, ABA selectively elevated Chl b by 40.8% without significantly affecting other pigments, suggesting a priming effect on the peripheral light-harvesting antenna under non-stressful conditions [21]. This pigment-partitioning strategy diverges from cold-acclimation mechanisms reported in cold-tolerant pomegranate (*Punica granatum*) cultivars [22], where sustained chlorophyll retention supports continued carbon assimilation.

The apparent mismatch between extractable pigment content and visible leaf health warrants explicit comment. First, the increase in total chlorophyll from the ambient control (approximately 1.07 mg·g^−1^ FW) to the LT group (approximately 1.22 mg·g^−1^ FW) is modest, involves a cross-panel comparison that was not subjected to statistical testing, and we therefore do not interpret it as a treatment effect; the only pigment contrast under freezing that reached significance was the LT–ALT comparison itself. Second, extractable chlorophyll content is not a direct measure of photochemical competence: chlorophyll can be retained in tissues whose photosynthetic apparatus is no longer functional, as documented for non-functional stay-green phenotypes [23], and because chlorophyll degradation is enzyme-catalysed [19], it is expected to stall under sub-zero temperatures. In line with this expectation, the LT–ALT difference was confined almost entirely to chlorophyll a (7-fold, *p* = 0.007), the primary substrate of the chlorophyll-degradation pathway, whereas chlorophyll b did not differ between groups (*p* = 0.612); xanthophylls showed the same retention pattern (43-fold enrichment in LT leaves), indicating that retention extended across pigment classes subject to turnover. Third, pigment composition, rather than absolute content, distinguished the treatments: carotenoids were approximately 7-fold more abundant in ALT than in LT leaves (*p* = 0.010), so that the chlorophyll-to-carotenoid balance of ALT leaves remained far closer to that of healthy tissue. For the same reason, studies of freezing injury in woody evergreens, including *Rhododendron*, rely on electrolyte-leakage or chlorophyll-fluorescence measurements rather than on extractable pigment content as primary injury indices [24,25]. We therefore present the pigment data descriptively: the protective effect of ABA inferred in this study rests on the concordant evidence of visual injury scores, MDA content, osmoprotectant accumulation, and antioxidant enzyme activities, and not on pigment content itself.

### 4.3. Effect of Exogenous ABA on the Osmoregulatory System of R. obtusum ‘Fen Zhuanglou’

Osmoprotectants contribute to plant cold tolerance: their accumulation increases cytoplasmic solute concentration, which enhances water absorption and retention and prevents physiological dehydration under low-temperature stress [26]. In this study, ABA treatment at normal temperature promoted the accumulation of three osmoprotectants—SS, SP, and Pro—a finding consistent with previous studies on grape (*Vitis vinifera*) flower buds [27]. After cold stress, the contents of all three osmoprotectants significantly increased, and exogenous ABA application further enhanced SS accumulation. SS not only functions as an osmoprotectant but also acts as a substrate for energy conversion and a signaling molecule involved in cold response [28].

Regarding SP content, our results showed that ABA treatment at normal temperature significantly increased SP levels. However, under cold stress, SP content in the ABA-treated group was lower than in the cold control group. This may reflect intensified metabolic consumption at low temperature, under which SP is preferentially degraded to supply energy for essential processes, so that degradation exceeds synthesis.

Pro content showed significant increases under ABA treatment at normal temperature, consistent with findings in rice and Antarctic moss [18,29]; however, under cold stress, Pro content in the ABA-treated group was only marginally higher than in the cold control group (+2.15%, *p* > 0.05). This suggests that proline biosynthesis may not be the primary osmotic target of ABA in this species under these conditions. This contrasts with studies in rice and grapevine where ABA strongly induces proline accumulation. The lack of a significant proline response in *R. obtusum* ‘Fen Zhuanglou’ may reflect species-specific differences in osmotic adjustment strategies, or alternatively, proline may be preferentially allocated to non-leaf tissues under freezing stress—a hypothesis requiring tissue-specific metabolite profiling for validation.

### 4.4. Regulation of Antioxidant Enzyme System in R. obtusum ‘Fen Zhuanglou’ by Exogenous ABA

When plants are subjected to low-temperature stress, the redox balance within their cells is disrupted, leading to excessive accumulation of ROS and subsequent oxidative damage such as membrane lipid peroxidation [30]. The results of this study show that low-temperature stress caused a sharp decline in antioxidant enzyme activity in *R. obtusum* ‘Fen Zhuanglou’, with SOD, POD, and CAT activities decreasing by 58.3%, 68.9%, and 68.2%, respectively, indicating that −4 °C severely inhibited the normal function of the antioxidant enzyme system.

However, exogenous ABA treatment significantly alleviated the decline in antioxidant enzyme activity. In the ALT 5 d group, SOD, POD, and CAT activities were increased by 40.0%, 29.2%, and 35.2%, respectively, compared to the LT 5 d group, suggesting that exogenous ABA can enhance the cold tolerance of *R. obtusum* ‘Fen Zhuanglou’ by modulating antioxidant enzyme activity. This agrees with previous findings in winter rapeseed, Ligustrum lucidum, and pepper [6,31,32], where exogenous ABA enhanced antioxidant enzyme activity and mitigated cold-induced damage. Notably, enzyme activities in the low-temperature ABA-treated group remained significantly lower than those in the ambient-temperature ABA-treated group, indicating that −4 °C cold stress imposed strong physiological inhibition on *R. obtusum* ‘Fen Zhuanglou’.

### 4.5. The Alleviating Effect of Exogenous ABA on Membrane Lipid Peroxidation in R. obtusum ‘Fen Zhuanglou’

MDA is a product of membrane lipid peroxidation and is widely used as a biological indicator of oxidative damage in plants [33]. The results of this study showed that low-temperature stress at −4 °C significantly increased MDA content, with the MDA level in the LT 5 d group being 3.4-fold higher than that in the CK 5 d group. However, the ALT 5 d group exhibited a significant 23.5% reduction compared to the LT 5 d group, indicating that exogenous ABA partially alleviated membrane lipid peroxidation and slowed membrane damage. Even after ABA treatment, MDA levels remained significantly higher than those in the control at normal temperature, suggesting that the protective effect of ABA is limited under extreme low temperatures of −4 °C and cannot completely prevent membrane structural damage.

Limitations of the mechanistic interpretation: While we propose a resource-reallocation framework involving pigment remodelling, metabolic trade-offs, and antioxidant enzyme rescue, we acknowledge that several key parameters were not measured in this study: relative electrolyte leakage (REC), photosynthetic rate (Pn), chlorophyll fluorescence (Fv/Fm), ROS (H_2_O_2_, O^2−^), ascorbate peroxidase (APX), and glutathione reductase (GR). Therefore, our mechanistic conclusions should be interpreted as hypotheses requiring validation in future studies integrating physiological, biochemical, and molecular approaches. In addition, pigment contents were expressed on a fresh-weight basis; although all leaf samples were harvested at the same position and developmental stage, weighed immediately after harvest, and extracted in parallel, a potential contribution of differential tissue water content to the pigment contrast between groups cannot be fully excluded.

## 5. Conclusions

(1) An exogenous ABA concentration of 10 mg·L^−1^ was optimal for enhancing cold tolerance in *R. obtusum* ‘Fen Zhuanglou’. This concentration effectively alleviated cold injury symptoms (leaf necrosis, curling, and abscission) caused by low-temperature stress at −4 °C and maintained normal leaf morphology.
(2) The regulation of photosynthetic pigments in *R. obtusum* ‘Fen Zhuanglou’ by exogenous ABA exhibited both concentration specificity and temperature dependence. Under low-temperature stress, the contents of chlorophyll a and xanthophylls in the LT group were 7-fold and 43-fold higher than those in the ALT group, respectively, while carotenoid content in the ALT group was approximately 7 times higher than that in the LT group; under normal temperature conditions, only chlorophyll b content was significantly regulated by ABA.
(3) Exogenous ABA enhanced osmotic adjustment capacity in *R. obtusum* ‘Fen Zhuanglou’. Under low-temperature stress, treatment with the optimal ABA concentration significantly increased soluble sugar content by 6.05% compared to the low-temperature control group, but significantly decreased soluble protein content by 22.98%, while proline content increased by 2.15%, though not significantly.
(4) Exogenous ABA activated the antioxidant enzyme system in *R. obtusum* ‘Fen Zhuanglou’. Under low-temperature stress, the optimal ABA concentration significantly increased SOD, POD, and CAT activities by 40.0%, 29.2%, and 35.2%, respectively, compared to the low-temperature control group; however, these three enzyme activities remained significantly lower than those in the normal-temperature ABA-treated group.
(5) Exogenous ABA reduced membrane lipid peroxidation damage in *R. obtusum* ‘Fen Zhuanglou’ under low-temperature stress. Treatment with the optimal ABA concentration significantly decreased MDA content by 23.5% compared to the low-temperature control group, although MDA levels remained significantly higher than those in the normal-temperature control group.

Broader implications: These findings provide an evidence-based ABA application protocol (10 mg·L^−1^) for ornamental rhododendron production in cold regions. This resource-reallocation framework may extend to other ornamental woody species facing climate instability. Limitations: This study is limited by the lack of molecular validation (e.g., gene expression of chlorophyll catabolism enzymes and ABA signaling components) and the absence of photosynthetic functional measurements (e.g., Fv/Fm, gas exchange). The visual scoring system for optimal concentration screening, while practical, introduces subjectivity. Further studies should integrate chlorophyll fluorescence imaging, transcriptomic analysis of ABA-responsive genes, and field validation across multiple growing seasons to confirm the robustness of the 10 mg·L^−1^ threshold under fluctuating natural conditions.

## Data Availability

Data are contained within the article and Supplementary Materials.

## Supplementary Materials

The following supporting information can be downloaded at: https://www.mdpi.com/article/10.3390/plants15213457/s1, Figure S1: Experimental design and treatment timeline of exogenous ABA application under low-temperature stress; Table S1: Detailed statistical outputs for all pairwise comparisons of physiological indicators in *R. obtusum* ‘Fen Zhuanglou’.

## References

[1] Dong J., Wu Y.W., Dong Y., Pu R., Li X.J., Lyu Y.M., Bai T., Zhang J.L. (2024). Genome-wide identification of the *bHLH* gene family in *Rhododendron delavayi* and its expression analysis in different floral tissues. Genes.

[2] Shrestha N., Wang Z., Su X., Xu X., Lyu L., Liu Y., Dimitrov D., Kennedy J.D., Wang Q., Tang Z. (2018). Global patterns of *Rhododendron* diversity: The role of evolutionary history, climate, and topography. Glob. Ecol. Biogeogr..

[3] Ritonga F.N., Chen S. (2020). Physiological and molecular mechanism involved in cold stress tolerance in plants. Plants.

[4] Cutler S.R., Rodriguez P.L., Finkelstein R.R., Abrams S.R. (2010). Abscisic acid: Emergence of a core signaling network. Annu. Rev. Plant Biol..

[5] Li Y., Wang M., Guo T., Li S., Teng K., Dong D., Liu Z., Jia C., Chao Y., Han L. (2022). Overexpression of abscisic acid-insensitive gene ABI4 from Medicago truncatula, which could interact with ABA2, improved plant cold tolerance mediated by ABA signaling. Front. Plant Sci..

[6] Guo W.L., Chen R.G., Gong Z.H., Yin Y.X., Ahmed S.S., He Y.M. (2012). Exogenous abscisic acid increases antioxidant enzymes and related gene expression in pepper (*Capsicum annuum*) leaves subjected to chilling stress. Genet. Mol. Res..

[7] Xiang H.-T., Wang T.-T., Zheng D.F., Wang L.-Z., Feng Y.-J., Luo Y., Li R., Li Z.-J., Meng Y., Li W. (2017). ABA pretreatment enhances the chilling tolerance of a chilling-sensitive rice cultivar. Braz. J. Bot..

[8] Khaleghnezhad V., Popescu V.S., Yousefi A.R., Tavakoli A., Mastinu A. (2025). Abscisic acid effect on the alteration of the photosynthetic processes induced by thermal stress in *Dracocephalum moldavica* L.. Vegetos.

[9] Liu X.M. (2017). Evaluation of Cold Tolerance in *Rhododendron* Cultivars and the Effect of Exogenous ABA on Their Cold Tolerance. Master’s Thesis.

[10] Liang Q.Y., Pan M.J., Yin Y.Q., Wei J.Y., Shen F.K., Jia H.J., Tang X.L. (2023). Effects of exogenous ABA on physiological indexes of flue-cured tobacco seedlings under low temperature stress. J. Northeast Agric. Sci..

[11] Zhang Y., Fu X., Feng Y., Zhang X., Bi H., Ai X. (2022). Abscisic acid mediates salicylic acid induced chilling tolerance of grafted cucumber by activating H_2_O_2_ biosynthesis and accumulation. Int. J. Mol. Sci..

[12] Zeng Y.X., Qin Q.Q., Liu Y.H. (2024). Effects of nighttime light on photosynthetic pigments and secondary metabolism of *Ginkgo biloba* L. leaves during the discoloration period. Chin. J. Appl. Environ. Biol..

[13] Li C., Pan Y., Cui J., Lu X., Yu W. (2025). Mechanism of ABA in plants exposed to cold stress. Agronomy.

[14] Chinnusamy V., Ohta M., Kanrar S., Lee B.H., Hong X., Agarwal M., Zhu J.K. (2003). ICE1: A regulator of cold-induced transcriptome and freezing tolerance in Arabidopsis. Genes Dev..

[15] Ding Y., Li H., Zhang X., Xie Q., Gong Z., Yang S. (2015). OST1 kinase modulates freezing tolerance by enhancing ICE1 stability in Arabidopsis. Dev. Cell.

[16] Kwak J.M., Mori I.C., Pei Z.M., Leonhardt N., Torres M.A., Dangl J.L., Bloom R.E., Bodde S., Jones J.D.G., Schroeder J.I. (2003). NADPH oxidase AtrbohD and AtrbohF genes function in ROS-dependent ABA signaling in Arabidopsis. EMBO J..

[17] Mittler R., Blumwald E. (2015). The roles of ROS and ABA in systemic acquired acclimation. Plant Cell.

[18] Seo S., Ahn Y., Kim K., Lee J.H., Min K., Lee H. (2025). Exogenous abscisic acid enhances freeze-thaw stress tolerance in Antarctic moss *Sanionia uncinata* through coordinated antioxidant defense and osmoprotectant accumulation. Cryobiology.

[19] Hörtensteiner S. (2006). Chlorophyll degradation during senescence. Annu. Rev. Plant Biol..

[20] Mirkovic T., Ostroumov E.E., Anna J.M., van Grondelle R., Govindjee, Scholes G.D. (2017). Light absorption and energy transfer in the antenna complexes of photosynthetic organisms. Chem. Rev..

[21] Travaglia C., Cohen A.C., Reinoso H., Castillo C., Bottini R. (2007). Exogenous abscisic acid increases carbohydrate accumulation and redistribution to the grains in wheat grown under field conditions of soil water restriction. J. Plant Growth Regul..

[22] Guan S.H., Chai Y.Q., Yang Y.L., Cui H.X., Zhang J. (2024). Effects of low temperature stress on photosynthetic characteristics and antioxidant capacity of pomegranate. Chin. Agric. Sci. Bull..

[23] Thomas H., Howarth C.J. (2000). Five ways to stay green. J. Exp. Bot..

[24] Lim C.C., Arora R., Townsend E.C. (1998). Comparing Gompertz and Richards functions to estimate freezing injury in *Rhododendron* using electrolyte leakage. J. Am. Soc. Hortic. Sci..

[25] Öquist G., Huner N.P.A. (2003). Photosynthesis of overwintering evergreen plants. Annu. Rev. Plant Biol..

[26] Roychowdhury R., Das S.P., Sarkar P., Khan Z., Kumar A., Sarker U., Sajeevan R. (2025). Physiological, biochemical and molecular signaling basis of cold stress tolerance in plants. Front. Plant Sci..

[27] Rubio S., Noriega X., Pérez F.J. (2019). Abscisic acid (ABA) and low temperatures synergistically increase the expression of CBF/DREB1 transcription factors and cold-hardiness in grapevine dormant buds. Ann. Bot..

[28] Huang X., Shi H., Hu Z., Liu A., Amombo E., Chen L., Fu J. (2017). ABA is involved in regulation of cold stress response in Bermudagrass. Front. Plant Sci..

[29] Cao X., Wu L., Wu M., Zhu C., Jin Q., Zhang J. (2020). Abscisic acid mediated proline biosynthesis and antioxidant ability in roots of two different rice genotypes under hypoxic stress. BMC Plant Biol..

[30] Mittler R. (2002). Oxidative stress, antioxidants, and stress tolerance. Trends Plant Sci..

[31] Zhang T., Kou X.E., Liu H.Z., Li Y.P., Zhang D.L., Zeng X.C., Xu Y.Z. (2023). Effects of exogenous ABA spraying on physiological characteristics of winter rapeseed Longyou 7 seedlings under low temperature. Bull. Agric. Sci. Technol..

[32] Yang Y., Zhang R., Duan X., Hu Z., Shen M., Leng P. (2019). Natural cold acclimation of *Ligustrum lucidum* in response to exogenous application of paclobutrazol in Beijing. Acta Physiol. Plant..

[33] Hodges D., DeLong J., Forney C., Prange R.K. (1999). Improving the thiobarbituric acid-reactive-substances assay for estimating lipid peroxidation in plant tissues containing anthocyanin and other interfering compounds. Planta.

