# Supplementary material for: Exogenous Abscisic Acid Enhances Freezing Tolerance in Rhododendron obtusum ‘Fen Zhuanglou’ by Stabilizing Photosynthetic Pigments, Promoting Osmolyte Accumulation, and Activating Antioxidant Defense
Source: Plants (Basel). 2026 Nov 5;15(21):3457. doi: 10.3390/plants15213457 (PMC13765908; doi:10.3390/plants15213457)
Supplement: Supplementary file 1 [file plants-15-03457-s001.zip › Supplementary Table S1.pdf]

Supplementary Table S1. Detailed statistical outputs for all pairwise comparisons of physiological indicators in *R. obtusum* 'Fen Zhuanglou'.

Category	Indicator	Comparison	Relative change	Test	df	*P* value
Photosynthetic pigments	Chl a	ALT 5 d vs. LT 5 d (−4 °C)	LT = 7.0 × ALT	t-test	4	0.007 **
	Chl b	ALT 5 d vs. LT 5 d (−4 °C)	no difference	t-test	4	0.612 ns
	Xanthophylls	ALT 5 d vs. LT 5 d (−4 °C)	LT = 43 × ALT	t-test	4	0.001 **
	Carotenoids	ALT 5 d vs. LT 5 d (−4 °C)	ALT = 7.0 × LT	t-test	4	0.010 *
	Chl a	A 5 d vs. CK 5 d (22 °C)	+29.5%	t-test	4	0.101 ns
	Chl b	A 5 d vs. CK 5 d (22 °C)	+40.8%	t-test	4	0.030 *
	Xanthophylls	A 5 d vs. CK 5 d (22 °C)	+23.3%	t-test	4	0.217 ns
	Carotenoids	A 5 d vs. CK 5 d (22 °C)	+3.7%	t-test	4	0.888 ns
Osmolytes	Soluble sugar (SS)	LT 5 d vs. CK 5 d	+349.21%	DMRT	8	<0.05 *
	Soluble protein (SP)	LT 5 d vs. CK 5 d	+200.97%	DMRT	8	<0.05 *
	Proline (Pro)	LT 5 d vs. CK 5 d	+351.40%	DMRT	8	<0.05 *
	Soluble sugar (SS)	A 5 d vs. CK 5 d	+159.36%	DMRT	8	<0.05 *
	Soluble protein (SP)	A 5 d vs. CK 5 d	+231.82%	DMRT	8	<0.05 *
	Proline (Pro)	A 5 d vs. CK 5 d	+84.91%	DMRT	8	<0.05 *
	Soluble sugar (SS)	ALT 5 d vs. LT 5 d	+6.05%	DMRT	8	<0.05 *
	Soluble protein (SP)	ALT 5 d vs. LT 5 d	−22.98%	DMRT	8	<0.05 *
	Proline (Pro)	ALT 5 d vs. LT 5 d	+2.15%	DMRT	8	>0.05 ns
Antioxidant enzymes	SOD	A 5 d vs. CK 5 d	+11.9%	DMRT	8	<0.05 *
	POD	A 5 d vs. CK 5 d	+10.9%	DMRT	8	<0.05 *
	CAT	A 5 d vs. CK 5 d	+3.0%	DMRT	8	>0.05 ns
	SOD	ALT 5 d vs. LT 5 d	+40.0%	DMRT	8	<0.05 *
	POD	ALT 5 d vs. LT 5 d	+29.2%	DMRT	8	<0.05 *
	CAT	ALT 5 d vs. LT 5 d	+35.2%	DMRT	8	<0.05 *
MDA	MDA	LT 5 d vs. CK 5 d	3.4 × CK	DMRT	8	<0.05 *
	MDA	ALT 5 d vs. CK 5 d	2.6 × CK	DMRT	8	<0.05 *
	MDA	ALT 5 d vs. LT 5 d	−23.5%	DMRT	8	<0.05 *

Note: For ambient-temperature pigment comparisons, Levene’s test confirmed homogeneity of variance (Chl a, $P = 0.225$; Chl b, $P = 0.504$; xanthophylls, $P = 0.929$; carotenoids, $P = 0.085$). Baseline stability checks (CK 0 d vs. CK 5 d at 22 ° C) showed no significant changes for all indicators other than MDA ($P > 0.05$); MDA increased modestly but significantly ($P < 0.01$), although its absolute content remained low.
